# Accidental Ingestion of Intoxications (Drugs and Nondrug Materials) Among Pediatric Patients: A Multicenter Study in Bahrain Royal Medical Services Hospitals

**DOI:** 10.1155/jt/2093893

**Published:** 2025-09-09

**Authors:** Noora Aljalahma, Lana Alabbasi, Heba Alkoheji, Hend Almahmood, Abdulrahman Aldoseri, Fatema Husain, Maryam Abdul Jabbar, Nawara Aljasim, Sara Dawood, Raafat Hammad

**Affiliations:** ^1^Department of General Surgery, Royal Medical Services, Bahrain Defence Force Hospital, Riffa, Bahrain; ^2^Department of Internal Medicine, Royal Medical Services, King Hamad University Hospital, Busaiteen, Bahrain; ^3^Department of Internal Medicine, Royal Medical Services, Bahrain Defence Force Hospital, Riffa, Bahrain; ^4^Department of Pediatric Hematology Oncology, Royal Medical Services, Bahrain Oncology Center, Busaiteen, Bahrain; ^5^Department of Pediatric Cardiology, Royal Medical Services, Mohammed Bin Khalifa Bin Salman Al Khalifa Cardiac Center, Awali, Bahrain; ^6^Salmaniya Medical Complex, Manama, Bahrain; ^7^Department of Medical Oncology, Royal Medical Services, Bahrain Oncology Center, Busaiteen, Bahrain; ^8^Department of Pediatrics, Royal Medical Services, Bahrain Defence Force Hospital, Riffa, Bahrain

**Keywords:** accidental ingestion, Bahrain, Bahrain defence force, intoxications, King Hamad University, paediatrics, Royal Medical Services Hospitals

## Abstract

The presence of accidental ingestion of toxins in children is a widespread and preventable issue. Even though most children are asymptomatic, prompt presentation and management have a significant impact on mortality. Each toxin has a different presentation and complication, with some resulting in organ injury. It is essential to highlight the reasons for accidental ingestions to prevent fatal consequences. This retrospective cross-sectional study was done in Royal Medical Services hospitals, Bahrain Defence Force Hospital, and King Hamad University Hospital in the Kingdom of Bahrain. Our study evaluates the incidence of accidental consumption of drugs and nonpharmacological toxins in children under the age of 14 years old who were admitted from the year 2022-2023. A total of 95 children were included, and the primary outcome was to recognize the most common toxin ingested and estimate the total number of accidental ingestions in two major hospitals in the Kingdom of Bahrain. The most common occurrence was observed in children aged between 2 and 3 years. Medications were the most prevalent type of intoxication, representing 55.6% of cases in 2022 and 56.1% in 2023. Among the 95 children, 48 were symptomatic, while 47 were asymptomatic. The highest rates of asymptomatic cases were observed in children who ingested medications. The average time between toxins ingestion and presentation to the emergency department was 90 min. All patients were discharged home in good condition, with zero mortality rate. Despite the fact that our research reported zero mortality, accidental ingestion of intoxications remains a serious and significant matter. Raising community awareness and sensibility through social and medical campaigns are highly encouraged, to ensure that not only caregivers but also people of all age groups recognize these urgent incidents promptly and respond appropriately. Additionally, intensified preventive measures involving closer supervision of minors and educating children themselves about the risks can also decrease the number of voluntary intoxication cases.

## 1. Introduction

Accidental ingestion is a worldwide and preventable issue in the pediatric population, leading to serious consequences or sometimes even death. A retrospective study done on accidental poisoning in children conducted by Aldeen HA in Salmaniya Medical Complex (SMC) in the Kingdom of Bahrain found over 600 cases requiring admission, with 146 being symptomatic upon presentation. Moreover, 2 cases occurred due to rat poisoning and orphenadrine. Among the selected population, children under the age of two ingested mainly hydrocarbons. In contrast, those over the age of two had a higher incidence of medication ingestion found at their homes. The majority of the children affected were males [1]. In 2021, a study performed by Husain A in the Bahrain Defence Force (BDF) Hospital affirmed that among the 88 patients included, most were below the age of 2 years, and no deaths were documented in the study. Additionally, the most ingested substance was found to be medications, antihypertensives in particular. Moreover, no gender differences were noted when it came to accidental ingestion as compared to Aldeen HA [2].

A study by Nistor et al. in “St Mary” hospital from Iasi, northeast Romania, found that children from ages 2-3 years were known to ingest medications such as nonsteroidal anti-inflammatory drugs (NSAIDs) and paracetamol, while those aged 0-1 year ingested nonpharmacological toxins, particularly household products. Patients who ingested NSAIDs suffered from gastrointestinal symptoms without serious consequences. On the contrary, paracetamol ingestion led to severe liver dysfunction, but overall, they survived by receiving adequate management with N-acetylcysteine [3]. A multicentric study done in Sri Lanka from 2007 to 2014 included over 36 hospitals and found that the predominant age group was less than five years of age from the recruited 410 patients between nine months and 12 years of age. Males were the highest affected gender group, with a range of 50.9% [4].

Additionally, paracetamol remains one of the most frequent drugs accidently ingested. Interestingly, due to this country's high dengue fever rates, ibuprofen is not given. Thus, it was not categorized as a common medication to be accidently ingested. Over 61 patients presented were asymptomatic, while symptomatic patients suffered from neurological symptoms such as vertigo, dizziness, and drowsy [4].

In 2023, Isa H conducted a retrospective study in SMC Bahrain on accidental ingestion of foreign bodies and harmful materials in children. Most children were between the ages of 2-3 years, likely due to being in the oral phase, thus prone to ingesting objects while crawling and playing. Similarly to Husain A's data, the prevalence of males ingesting toxins was significantly higher compared to females in this study. A total of 86/150 children were symptomatic upon presentation. This can be attributed to the differences in the time from ingestion to presentation and the type, shape, and amount of the ingested material [5].

Tobaiqy et al. stated that acute poisoning may differ from region to region due to socioeconomic status, culture, and familial education. Medications and cleaning products were mainly ingested in developed countries while developing countries had a higher incidence of pesticides and kerosene ingestion [6]. As per Nistor et al., rural areas are more at risk of intoxication than urban areas because the products being used are usually found in plastic bottles, therefore attracting the attention of children [3].

Another interesting study conducted in Korea focused on the importance of the drug formula in accidental ingestion. Cases of capsules and tablets (TAC) were more common to be accidently ingested compared to a drug that is in a syrup formula. Medications in the form of TAC, such as NSAIDs, also illustrated higher cases of hospitalization, particularly in infants. Medications in the form of syrup, including antihistamines, also have less severe outcomes due to the safety profile of such medications [7].

Another reason for accidental ingestions would be errors in dosing by the parents, especially for syrup medications. Thus, being cautious when providing accurate doses will lower future risks of accidental intoxication. It is also essential for healthcare providers to guide caregivers on first-line management when accidents occur, such as explaining red flags and highlighting the importance of prompt presentation to the nearest hospital [8]. Providing parents with a good history of the type of ingestion, time of ingestion, doses, and route will also aid in diagnosing and correctly managing cases [9].

This study aims to determine the number, types, and presenting symptoms of accidental ingestion-related intoxication among the pediatric population up to 14 years old, presenting to the Royal Medical Services (RMS) Hospitals in Bahrain, including BDF Hospital and King Hamad University Hospital (KHUH), respectively. To promote safety awareness, improve parental education, and strengthen emergency preparedness among healthcare providers.

## 2. Materials and Methods

In this retrospective cross-sectional study that was conducted in RMS Hospitals: BDF Hospital and KHUH, we evaluated the occurrence of accidental ingestion of intoxications (drugs and nonpharmacological products) among pediatric population from the year 2022–2023. Children under the age of 14 years admitted to the hospital's emergency unit due to accidental ingestion of toxic substances were included in this study. Children with incomplete data and those with foreign body ingestion or poisoning through food ingestion or with allergic reactions due to insect bites were excluded from the study. Approval of the study was obtained from institutional research and ethical board. Electronic medical records of the patients were used to collect data retrospectively. Variables included were demographics (age and gender), intoxications, symptoms, time of ingestion (most common time children consumed the material), the time of presenting with symptoms (window from ingesting to coming to hospital), number of mortalities (if any), and caused by which toxin. The primary outcome was identifying the most common material to be ingested accidently in children. The secondary outcome was to raise awareness in the country and to quantify the number of cases of accidental ingestion around the two hospitals.

### 2.1. Statistical Analysis

Continuous variables were represented as median (interquartile range), whereas categorical variables were represented as frequencies and percentages. SPSS (Version 26.0) was used to conduct all analyses.

## 3. Results

**Figure 1:**
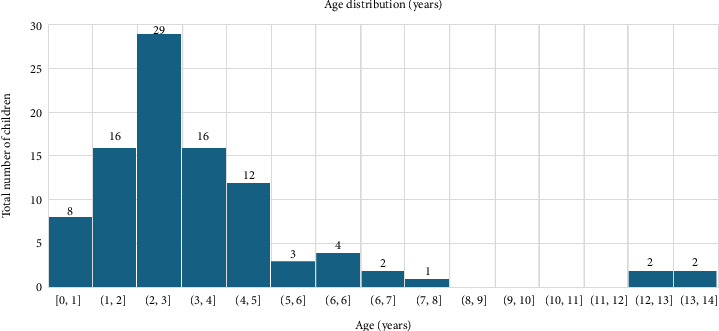
Children's age distribution (years), represented as frequencies.

The analyses included a total of 95 children who had an accidental ingestion of intoxication from the years 2022–2023; 59 were from BDF hospital, and 36 were from KHUH. Children's ages ranged from 0.1–13.8 years old, with a median and interquartile range of 2.7 (1.9–4). Children's baseline characteristics are represented in Table 1 as frequencies and percentages. Children's age distribution is illustrated in Figure 1.

**Figure 2:**
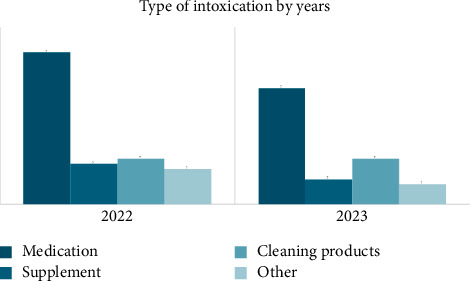
Annual comparison of intoxication type among children between 2022 and 2023, represented as frequencies and percentages *N* (%).

A total of 29 children aged between 2-3 years had the highest frequency of accidental ingestion of intoxications among other ages, as shown in Figure 1. An annual comparison of intoxication types among children between 2022 and 2023 is represented in Figure 2.

Medications were the most prevalent type of intoxication in both years, accounting for 55.6% in 2022 and 56.1% in 2023. Cleaning products were the second most common type of intoxication, with a total number of 9 children in both years.

**Figure 3:**
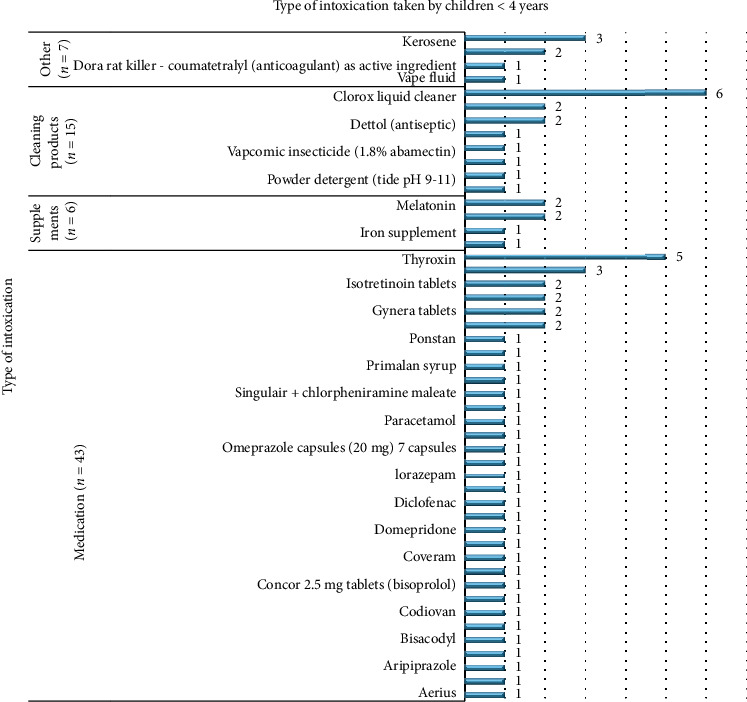
Distribution of types of intoxication taken by children < 4 years, represented as frequencies.

Data were further divided into two age groups, below 4 years, and 4 years and above, to additionally show examples of each intoxication category. Although medications were the mostly ingested intoxication in both age groups, Clorox Liquid cleaner was the most ingested product by children younger than 4 years among all intoxication types as mentioned in Figure 3.

**Figure 4:**
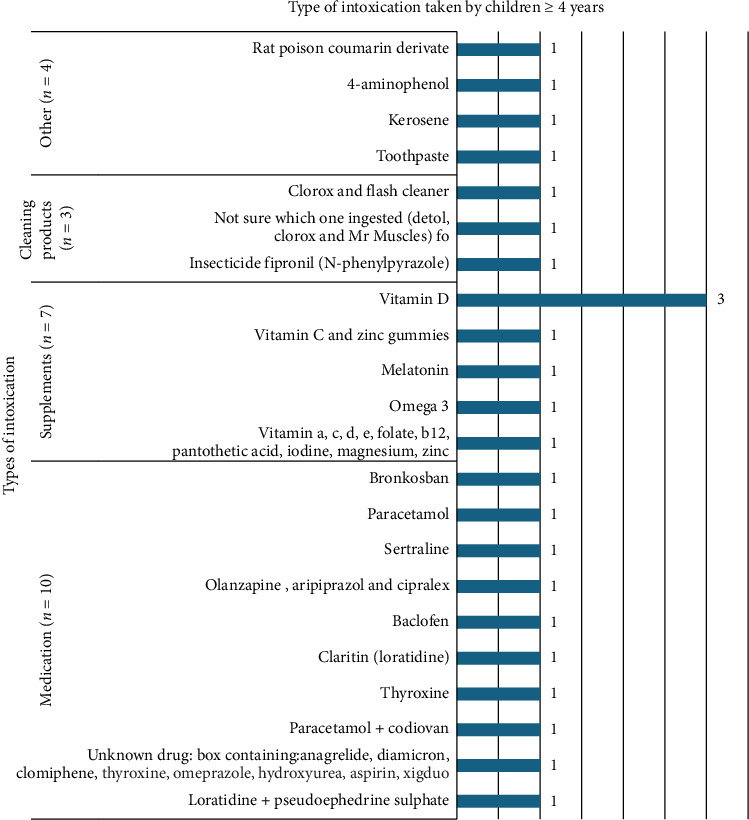
Distribution of types of intoxication taken by children ≥ 4 years, represented as frequencies.

Vitamin D supplement was the most ingested substance in children aged 4 years and older as illustrated in Figure 4.

Symptoms associated with types of intoxication are represented in Table 2. Children who ingested medications had the highest rate of being asymptomatic or experiencing drowsiness, with percentages of 56.6% and 20.8%, respectively. 61.5% of children who ingested supplements were asymptomatic. Children who ingested cleaning products experienced symptoms such as vomiting, abdominal pain, and coughing with 44.4%, 16.7%, and 33.3%, respectively.

**Figure 5:**
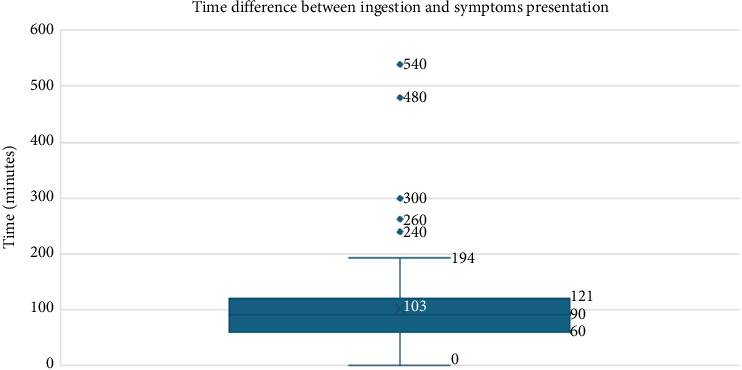
Time difference between ingestion and symptoms presentation in minutes. Two children had an extreme value of 2880 and 7200 minutes, which were not included in this figure.

The time difference between ingestion and symptom presentation is illustrated in Figure 5. The median time and interquartile range between children's ingestion and symptom presentation were 90 min (60–121). Two children had an extreme value of 2880 and 7200 minutes, which were not included in this figure.

The mortality rate was reported as zero, with all patients being discharged in stable conditions.

## 4. Discussion

Accidental poisoning is a worldwide health issue among children and adolescents, with a mortality rate reaching 5% [2]. The pattern of poisoning, however, remains variable based on sociocultural factors [4]. Similarly, our data have shown that medication ingestion was the most prevalent from other forms of intoxication, with 55.6% of cases in 2022 and 56.1% in 2023. A 2021 Bahraini study conducted by Husain et al. also revealed that 76.1% of pediatric patients ingested medications, while 23.9% ingested nondrug substances [2]. It is important to note that the majority of children in our study remained asymptomatic (56.6%) following ingestion of intoxications such as medication ingestion. This is supported by other studies such as Dayasiri K [4]. However, 20.8% of children in our study experienced drowsiness with medication ingestion, while cleaning products induced vomiting (44.4%), abdominal pain (16.7%), and coughing (33.3%). Neurological manifestations were also mainly seen in other reports [4].

Cleaning products were the second most common type of intoxication found in our data. These caustic agents are most dangerous as they can lead to severe esophageal burns [10]. Fortunately, the products have only induced GI manifestations with no further complications. Thus, it is essential for caregivers to carefully select, store, and inform children regarding the dangers of home supplies. However, this can be challenging as most children are affected during the sensorimotor developmental stage or oral phase, where exploring objects in their surroundings is common [5]. The average age of children impacted in our data was between 2 and 3 years (total *n* = 29), consecutive to Aldeen et al., with 76.5% of children being under 3 years of age in the Kingdom of Bahrain [1]. Moreover, the prevalence of children of the male gender was slightly higher than that of females (51.6% vs. 48.4%). A similar male to female ratio was noted in Aldeen et al., which was 1.6:1 [1].

According to Vilaça et al., most of the accidental ingestions were among the age group below 4 years, accounting mainly for medications and cleaning products [11]. Similarly, in our study, children below 4 years also had medications (60.5%) and cleaning products (21.1%) as commonly ingested intoxication.

Thomas et al. investigated the presentation of intoxicated patients to the emergency department. Their results showed that the median interval between poisoning and presentation to emergency departments was 120 min [12]. Our results reported that the time difference between children's toxication and presentation to the emergency department was 90 min. Early presentation following accidental ingestion increases the effectiveness of treatment, specifically gastrointestinal decontamination [13]. A late presentation of more than 1 hour from the time of ingestion to the hospital causes a poor prognosis [9]. There are various reasons why caregivers presented late to the hospital following their child's accidental ingestion. A multicentric study done in Sri Lanka found that 14 cases presented to the ED over 6 h from the time of ingestion due to unsureness of the need for emergency treatment, while 6 cases were due to lack of awareness that the child was poisoned until the symptoms appeared [4]. However, delayed hospital presentations of over 2 h were also detected in the cities of Nigeria, such as Ekiti and Jos [14]. Thus, we must also consider the privilege of nearby hospitals in a small country like Bahrain compared to larger countries.

The mortality rate of accidental ingestion of substances in children depends widely on the type of ingestion. Fortunately, no mortality was reported in our study. Overall, the mortality rate is low. The rate of deaths in children reported in a study in Iran was 0.3%, with another study showing 1.2% [15, 16]. Additionally, in a study in Northeast Romania, a rate of 0.62% was reported [3]. The type of substance ingested significantly impacts the patient's outcome, influencing the mortality rate. For instance, it has been reported by Narges et al. [15] that the most common cause of mortality was opioids such as methadone while Nistor et al. found that nonmedication intoxications are associated with the highest risk of fatality [3].

The color and texture of medications and supplements can induce attraction in children, causing this rise in intoxication. Parental awareness regarding the risks, proper storage, and vigilance is essential to prevent accidental poisoning [4]. The prevalence of cleaning products being ingested over the years has risen from 16.7% to 22% in our data. According to the World Health Organization and the United Nations Children's Fund, the substitution of potentially toxic domestic products for analogous and benign ones can reduce the chances of accidental intoxication [11]. Studies conducted by Kohli et al. and Polasa et al. have all demonstrated that accidental poisoning in children was due to lack of adult supervision, with a limited understanding of the storage of items, as well as the children's eagerness in exploring their surrounding and the tendency to put objects in their mouth [17–19].

### 4.1. Limitations

Indeed, there are certain limitations to be considered in our study. Initially, the study sample was small (95 patients), considering that we included two years (2022 and 2023) and two tertiary hospitals in the Kingdom of Bahrain. The number of days of hospital stay was not mentioned in the medical records; it would be beneficial to know which intoxication might lead to a shorter or longer hospital stay. Our main focus was on the number of patients in RMS: BDF and KHUH. However, we could have included SMC (which is not part of the RMS hospitals), another tertiary hospital in Bahrain, that could have added more to our data which may be considered in future research.

## 5. Conclusion

In conclusion, accidental ingestion of medications was the most prevalent in our study, which is similarly acknowledged in other studies worldwide. In children under 4 years of age, Clorox liquid cleaner was the most common toxic agent ingested. On the other hand, children who are 4 years and older most frequently ingested Vitamin D.

Intoxication among children remains an important issue to this day. Thankfully, as reported by our data, no mortalities have been declared from the accidental ingestions. Nevertheless, that does not make the issue any less significant.

We suggest doing medical and social campaigns to raise awareness in society and ensure that adults and people of different ages are educated about this matter. Moreover, it is crucial to ensure that caregivers are first aid certified or educated to have the ability to recognize, report, and manage these incidents, as early recognition and eliminating delay make a considerable difference in the hospital course and prognosis.

Subsequently, a long-term study should be conducted to evaluate the effectiveness of these protective and educational measures in our society.

## Figures and Tables

**Table 1:** Children's baseline characteristics represented as frequencies and percentages *N* (%).

Characteristics	*N* (%)
Gender:	
Male	49 (51.6)
Female	46 (48.4)
Type of intoxication:	
Medications	53 (55.8)
Cleaning products	18 (18.9)
Supplements	13 (13.7)
Other	11 (11.6)
Symptoms:^‡^	
Asymptomatic	47 (49.5)
Vomiting	19 (20.0)
Drowsy	16 (16.8)
Abdominal pain	10 (10.5)
Cough	8 (8.4)
Vomiting (induced)	5 (5.3)
Difficulty breathing	4 (4.2)
Diarrhea	4 (4.2)
Throat pain	2 (2.1)
Cyanosis	2 (2.1)
Fever	2 (2.1)
Drooling	2 (2.1)
Fatigue	1 (1.1)
Hyperactive	1 (1.1)
Belching (burping)	1 (1.1)
Rash	1 (1.1)
Palpitations	1 (1.1)
Tremor	1 (1.1)
Burn mouth sensation	1 (1.1)
Choking	1 (1.1)
Gagging	1 (1.1)
Chest heaviness	1 (1.1)
Decrease oral intake	1 (1.1)
Paleness (pallor)	1 (1.1)

^‡^Some children had more than one symptom.

**Table 2:** Symptoms associated with type of intoxication, represented as frequencies and percentages *N* (%).

	*N*	Type of intoxication			
		Medication	Supplements	Cleaning products	Other
Symptoms:					
Asymptomatic	47	30 (56.6)	8 (61.5)	5 (27.8)	4 (36.4)
Vomiting	19	6 (11.3)	1 (7.7)	8 (44.4)	4 (36.4)
Drowsy	16	11 (20.8)	2 (15.4)	1 (5.6)	2 (18.2)
Abdominal pain	10	4 (7.5)	1 (7.7)	3 (16.7)	2 (18.2)
Cough	8	1 (1.9)	0 (0.0)	6 (33.3)	1 (9.1)
Vomiting (induced)	5	3 (5.7)	0 (0.0)	1 (5.6)	1 (9.1)
Difficulty breathing	4	0 (0.0)	0 (0.0)	2 (11.1)	2 (18.2)
Diarrhea	4	2 (3.8)	1 (7.7)	1 (5.6)	0 (0.0)
Throat pain	2	0 (0.0)	0 (0.0)	1 (5.6)	1 (9.1)
Cyanosis	2	0 (0.0)	1 (7.7)	0 (0.0)	1 (9.1)
Fever	2	1 (1.9)	0 (0.0)	0 (0.0)	1 (9.1)
Drooling	2	0 (0.0)	0 (0.0)	1 (5.6)	1 (9.1)
Fatigue	1	1 (1.9)	0 (0.0)	0 (0.0)	0 (0.0)
Hyperactive	1	1 (1.9)	0 (0.0)	0 (0.0)	0 (0.0)
Belching (burping)	1	0 (0.0)	0 (0.0)	1 (5.6)	0 (0.0)
Rash	1	0 (0.0)	0 (0.0)	1 (5.6)	0 (0.0)
Palpitations	1	1 (1.9)	0 (0.0)	0 (0.0)	0 (0.0)
Tremor	1	1 (1.9)	0 (0.0)	0 (0.0)	0 (0.0)
Burn mouth sensation	1	1 (1.9)	0 (0.0)	0 (0.0)	0 (0.0)
Choking	1	0 (0.0)	0 (0.0)	1 (5.6)	0 (0.0)
Gagging	1	0 (0.0)	0 (0.0)	1 (5.6)	0 (0.0)
Chest heaviness	1	1 (1.9)	0 (0.0)	0 (0.0)	0 (0.0)
Decrease oral intake	1	0 (0.0)	0 (0.0)	1 (5.6)	0 (0.0)
Paleness (pallor)	1	1 (1.9)	0 (0.0)	0 (0.0)	0 (0.0)

## Data Availability

Data are openly available in a public repository that issues datasets with DOIs.

## References

[1] Aldeen H. A., Khan I. M., Al-madani R. (1999). *Accidental Poisoning in Children in Bahrain*.

[2] Husain A. A., Alkhudur D. A., Jadah R. H. S. (2021). *Clinical Profile of Acute Accidental Ingestions in Pediatric Populations*.

[3] Nistor N., Frasinariu O. E., Rugină A., Ciomaga I. M., Jităreanu C., Ştreangă V. (2018). Epidemiological Study on Accidental Poisonings in Children from Northeast Romania. *Medicine (Baltimore)*.

[4] Dayasiri K., Jayamanne S. F., Jayasinghe C. Y. (2020). Accidental and Deliberate self-poisoning with Medications and Medication Errors Among Children in Rural Sri Lanka. *Emergency Medicine International*.

[5] Isa H. M., Aldoseri S. A., Abduljabbar A. S., Alsulaiti K. A. (2023). Accidental Ingestion of Foreign bodies/harmful Materials in Children from Bahrain: a Retrospective Cohort Study. *World Journal of Clinical Pediatrics*.

[6] Tobaiqy M., Asiri B. A., Sholan A. H. (2020). Frequency and Management of Acute Poisoning Among Children Attending an Emergency Department in Saudi Arabia. *Pharmacy*.

[7] Ko Y., Jeon W., Choi Y. J., Yang H., Lee J. (2021). Impact of Drug Formulation on Outcomes of Pharmaceutical Poisoning in Children Aged 7 Years or Younger: a Retrospective Observational Study in South Korea. *Medicine (Baltimore)*.

[8] Babić Ž., Kordić N. B., Rešić A., Turk R. (2021). Characteristics of Unintentional Ingestion of Oral Non-steroidal Anti-inflammatory Drugs and Analgesics in Preschool Children. *Archives of Industrial Hygiene and Toxicology*.

[9] Molla Y. M., Belachew K. D., Ayehu G. W., Teshome A. A. (2022). Acute Poisoning in Children in Ethiopia: a cross-sectional Study. *Scientific Reports*.

[10] Lee J., Fan N. C., Yao T. C. (2019). Clinical Spectrum of Acute Poisoning in Children Admitted to the Pediatric Emergency Department. *Pediatrics & Neonatology*.

[11] Vilaça L., Volpe F. M., Ladeira R. M. (2020). Accidental Poisoning in Children and Adolescents Admitted to A Referral Toxicology Department of A Brazilian Emergency Hospital. *Revista Paulista de Pediatria*.

[12] Thomas S. H., Bevan L., Bhattacharyya S. (1996). Presentation of Poisoned Patients to Accident and Emergency Departments in the North of England. *Human & Experimental Toxicology*.

[13] Gökalp G., Nalbant T., Bıcılıoğlu Y. (2024). The Insidious Enemy of the Liver: the Situation in Childhood Acetaminophen Poisoning and Early N-AC Treatment. *Pediatric Emergency Care*.

[14] Areprekumor T. E., Joboy-Okei E., Amadin N. O., Kalu S. U. (2024). Patterns and Clinical Outcomes of Childhood Poisoning Presenting to a Children’s Emergency Department in Yenagoa, Nigeria: a 10-year Retrospective Study. *BMJ Paediatrics Open*.

[15] Gholami N., McDonald R., Farnaghi F., Hosseini Yazdi M., Zamani N., Hassanian-Moghaddam H. (2022). Fatal Outcome in Acutely Poisoned Children with Hospitalization: a 10-Year Retrospective Study from Tehran, Iran. *Pediatric Emergency Care*.

[16] Tarhani F., Nezami A., Heidari G., Hosseinizadeh-Salavati N. (2022). Epidemiological Study of Acute Unintentional Poisoning Among Children in Iran. *Drug Research*.

[17] K S. H., Singi Y., V C., Dabhi D. (2022). A Study on the Profile of Poisoning in the Paediatric Population in a Tertiary Care Teaching Hospital of Chitradurga Region. *Cureus*.

[18] Kohli U., Kuttiat V. S., Lodha R., Kabra S. K. (2008). Profile of Childhood Poisoning at a Tertiary Care Centre in North India. *Indian Journal of Pediatrics*.

[19] Polasa R., Sirangi M., Kagitapu S. (2016). Childhood Accidental Poisoning in South India. *Journal of Dental and Medical Sciences*.

